# Rare Presentation of Cutaneous Metastasis in Breast Carcinoma

**DOI:** 10.7759/cureus.76512

**Published:** 2024-12-28

**Authors:** Ramita Mukherjee, Brijesh k Singh, Hemanga Bhattacharjee, Rajinder Parshad

**Affiliations:** 1 Surgical Disciplines, All India Institute of Medical Sciences, New Delhi, IND; 2 Surgery, All India Institute of Medical Sciences, New Delhi, IND

**Keywords:** breast cancer, cutaneous, eyelid, metastasis, recurrence

## Abstract

Recurrence beyond the second year of diagnosis and metastasis to the skin and eyelids are rare occurrences in breast cancer. When cutaneous metastases present without local recurrence, they pose a significant diagnostic challenge. Here, we describe a case of breast cancer that recurred 16 years after the initial treatment, with the only indication of recurrence being unusual skin and eyelid lesions.

## Introduction

Breast cancer most commonly recurs within the first two years after diagnosis, with late relapses being uncommon [1]. The site of recurrence significantly impacts prognosis, as local skin recurrence (LSR), distant cutaneous metastasis (DCM), and eyelid metastasis (EM) are rare and associated with poor survival [2-5]. Cutaneous lesions pose a diagnostic challenge in the clinical setting. Here, we describe a case of breast cancer recurrence 16 years post-treatment, manifesting as metastasis to the eyelid and skin (rare sites of late metastasis).

## Case presentation

**Figure 1:**
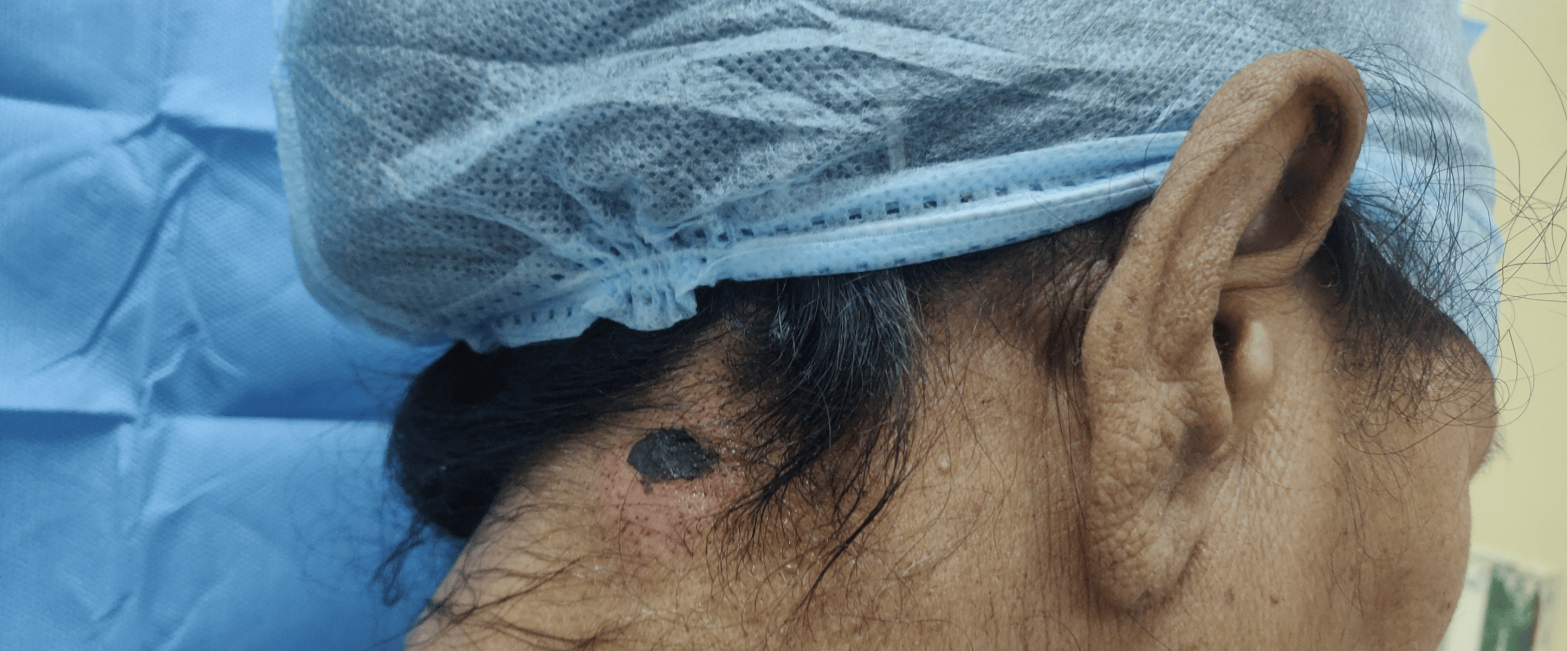
Eschar-like lesion on the right side of the nape of the neck.

**Figure 2:**
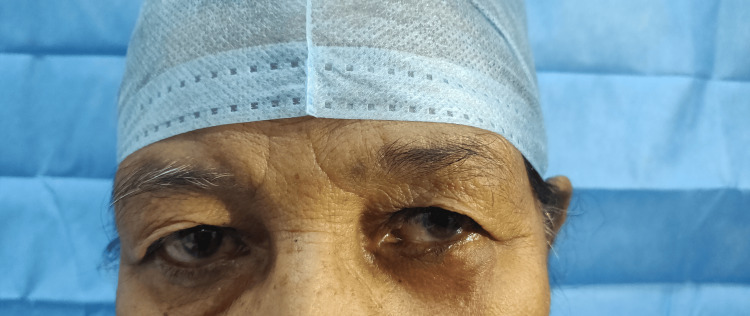
Skin-colored nodule on the left lower eyelid.

A 70-year-old woman presented with two eschar-like lesions on the right side of her nape (Figure 1) and a small, skin-colored nodule on the left lower eyelid (Figure 2), which had been present for three months. Both skin and eyelid lesions were non-itchy and painless. There was no history of fever, trauma, allergy, or any medication use. Her medical history included a diagnosis of early left-sided breast cancer (T2N0M0 ER 8/8, PR 8/8, HER2/neu negative) at the age of 54. The breast lesion, measuring 3 x 2 cm, was treated with breast-conserving surgery and sentinel lymph node biopsy, which showed no cancerous involvement in any of the four nodes. Following surgery, she underwent radiotherapy and was also started on anastrozole. She had regular follow-ups and remained disease-free until presenting with skin and eyelid lesions 16 years after surgery. Her breast and systemic examinations were within normal limits.

**Figure 3:**
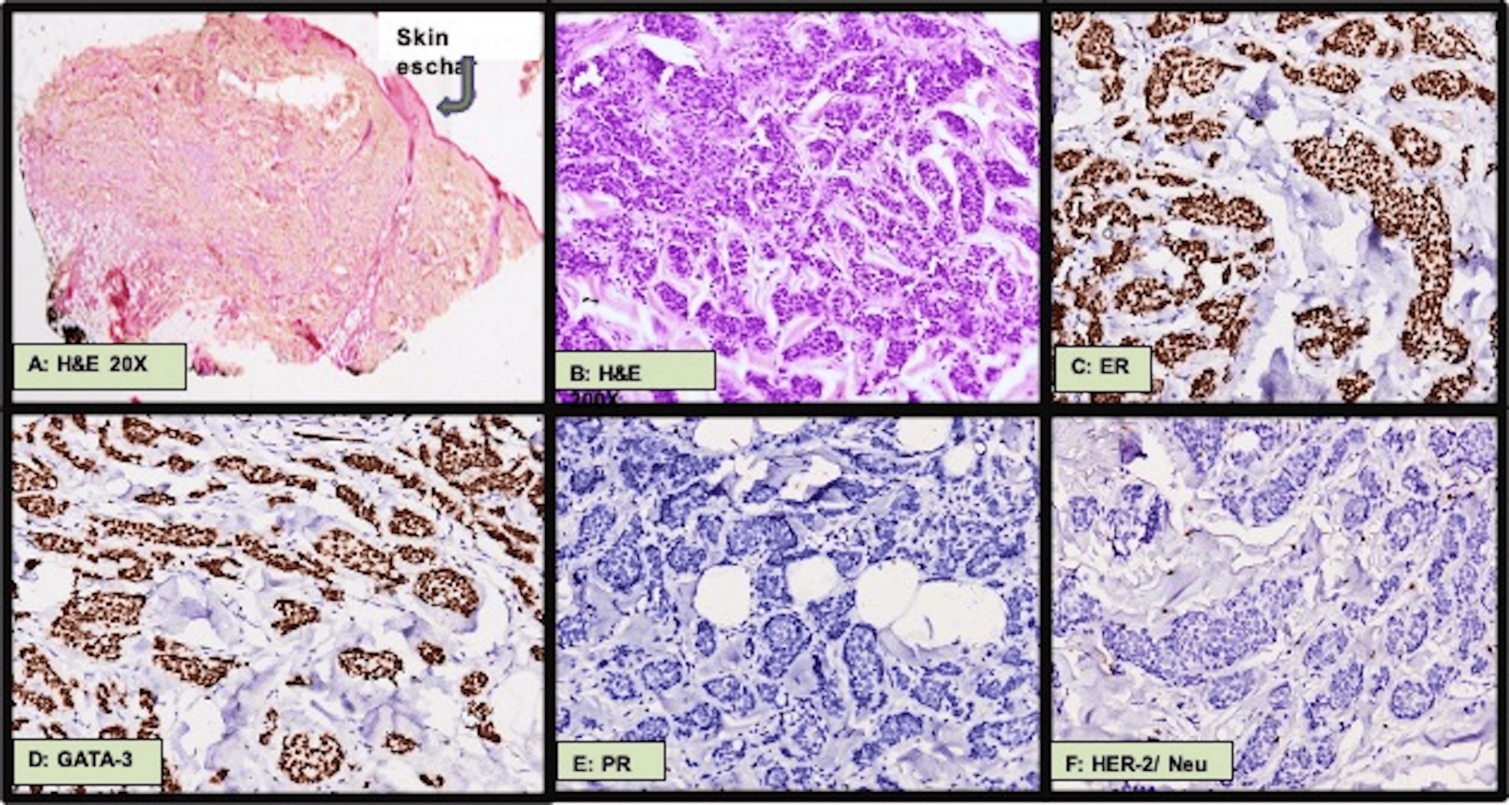
(A and B) Excision biopsy from neck lesion showing a skin-covered tissue with the sub-epithelium having metastatic adenocarcinoma. (C) Tumor cells immuno-positive for estrogen receptor (ER, 8/8 Allred score). (D) GATA-binding protein-3 (GATA-3) positivity. (E) Tumor cells immuno-negative for progesterone receptor (PR, 0/8 Allred score). (F) HER2/neu negative tumor tissues.

**Figure 4:**
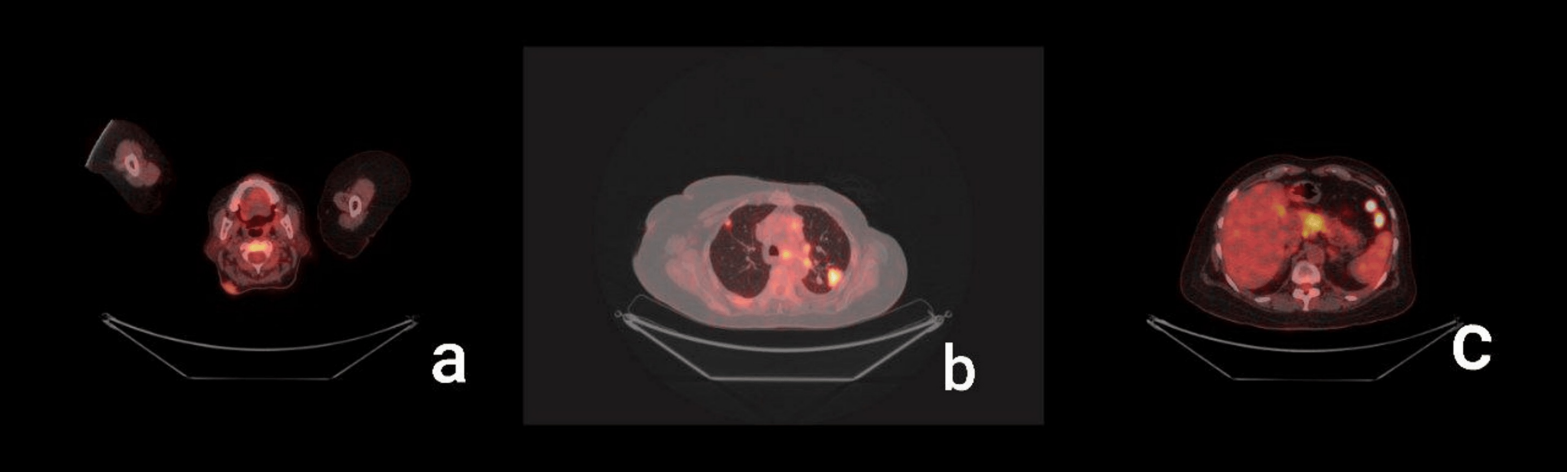
PET scan images showing FDG-avid uptake in the (a) neck, (b) lung, and (c) pancreas, suggestive of metastasis.

To exclude the recurrence of breast malignancy, mammography of both breasts was performed, yielding normal results. A biopsy of the neck lesion and fine needle aspiration (FNAC) of the eyelid swelling was planned after a dermatology consultation. Histopathological examination of the neck lesion revealed metastatic adenocarcinoma positive for ER and GATA-3 and negative for PR and HER2/neu (Figure 3). FNAC from the eyelid lesion also confirmed poorly differentiated adenocarcinoma. These findings indicated the need for further metastatic work-up. PET-CT showed an ill-defined FDG-avid tissue lesion measuring 2.2 x 2.4 cm with a speck of calcification at the head of the pancreas, and FDG-avid lytic sclerotic lesions in multiple vertebrae (cervico-dorso-lumbar), bilateral clavicles, scapulae, humeri, ribs, sacrum, and pelvic bones (Figure 4).

The patient was planned for palliative therapy. Unfortunately, she succumbed to the recurrence two months later. 

## Discussion

This report highlights the potential for recurrence in patients with ER+ early breast cancer despite receiving adjuvant endocrine therapy. The cumulative risk of recurrence in T2N0M0 cases has been reported to reach up to 19% over 5-20 years after completing endocrine therapy [6]. In our case, the ER+ status may have contributed to the delayed relapse.

The site of recurrence plays a significant role in survival outcomes. LSR and DCM are associated with advanced disease and poor prognosis. Isolated LSR, which occurs in 8% of breast cancer recurrences, is rare and linked to a higher rate of distant metastasis and lower survival rates compared to other recurrence sites (82%) [2]. DCM is the spread of cancer cells to the dermis and subcutaneous tissue without direct continuity to the primary tumor. It occurs in 0.7-10% of all invasive carcinomas, with breast cancer being the most common primary site after melanoma [7,8]. In a large retrospective study by Lookingbill et al., 30% of metastatic breast cancer patients had skin metastasis, with 66% involving the local site, 10% having DCM, and 24% patients showing both local and remote skin involvement [7]. Although breast cancer is the most common malignancy metastasizing to the skin, it remains rare, affecting only 2.8% of patients [1]. Skin lesions are the presenting sign in 3% of cases of metastatic breast cancer [9].

Skin metastasis from breast cancer most commonly presents as nodules (47%), with other common forms including alopecia neoplastica (12%), telangiectatic carcinoma (8%), melanoma-like lesions (6%), carcinoma erysipeloides (6%), subungual lesions (5%), carcinoma en cuirasse (4%), and zosteriform metastases (4%) as reported by De Giorgi [10]. Cutaneous involvement as a black eschar has not been previously reported.

In some cases, cutaneous metastases may be the first sign of an underlying breast malignancy or the first indication of relapse, as seen in the present case [11]. Navaratnam and Chandrasekharan reported a breast cancer relapse after 10 years of treatment, presenting as erythematous lesions on the right leg, initially misdiagnosed as dermatitis and intermittently treated with topical corticosteroids [12]. 

In our patient, the site of DCM was rare, affecting the nape of the neck and eyelids, in contrast to the more typical locations of the back and abdomen [10]. Metastatic eyelid lesions are rare, occurring in only 0.07%-0.3% of all eyelid lesions, with breast cancer being the most common primary source [5]. EM originates from breast cancer in 40% of cases, often presenting as diffuse eyelid swelling [5]. In a series of 13 patients, eyelid lesions preceded the diagnosis of primary breast cancer in five cases and signified the first sign of metastasis in four others [13]. Benson reported a case of nodular EM as the first sign of metastatic relapse five years after primary treatment of breast cancer [14]. Eyelid involvement in malignancy is typically associated with multi-organ metastasis, as seen in our patient. This case highlights the rarity of the involvement of the eyelid and nape of the neck as presenting signs of recurrent metastatic breast cancer. Metastatic skin lesions pose a diagnostic challenge and necessitate biopsy for definitive diagnosis.

## Conclusions

Cutaneous and eyelid metastatic recurrences in breast cancer patients can present in various forms. Skin metastasis often indicates advanced disease and poses a diagnostic challenge. These lesions require a biopsy for confirmation. Therefore, any patient with breast cancer presenting with suspicious skin lesions must undergo a thorough evaluation to rule out metastasis.
